# Time‐Related Burden of Treatment With Parenterally Administered Complement Inhibitors: A Mixed Methods Observational Study Exploring Experiences among Patients With Paroxysmal Nocturnal Hemoglobinuria

**DOI:** 10.1002/hsr2.72585

**Published:** 2026-06-19

**Authors:** David Dingli, Lynne Broderick, Avery A. Rizio, Sloan Rucker, Kaitlin LaGasse, Michelle K. Carty, Elise Burton, Shaquilla Gordon, Glorian P. Yen, Jincy Paulose, Anumaxine Geevarghese, Soyon Lee

**Affiliations:** ^1^ Mayo Clinic Rochester Minnesota USA; ^2^ IQVIA Durham North Carolina USA; ^3^ Jackson Heights New York City New York USA; ^4^ The Atlantic Beach Altantic Beach Florida USA; ^5^ Novartis Pharmaceuticals Corporation East Hanover New Jersey USA

## 1 Introduction

Paroxysmal nocturnal hemoglobinuria (PNH) is a rare, life‑threatening clonal hematopoietic stem cell disorder affecting approximately 12–13 per million individuals in the United States [1, 2, 3]. Caused by a somatic PIGA mutation, PNH results in deficiency of glycosylphosphatidylinositol‑anchored proteins on red blood cells, leading to complement‑mediated intravascular and extravascular hemolysis [2, 3].

Several parenterally administered complement inhibitors (PACIs) are approved in the US for treatment of PNH. Eculizumab and ravulizumab are administered via intravenous infusion by a medical professional at home or at a medical facility. Eculizumab infusions typically take 35 min and are administered every 2 weeks, while the duration of ravulizumab infusions, administered every 8 weeks, is dependent on the patient's weight [4, 5]. Pegcetacoplan is self‐administered at home, 2–3 times a week through subcutaneous infusions lasting 30–60 min [6].

Given the multiple treatment options available for patients with PNH, treatment decisions should be guided not only by efficacy, but also by how the treatment, including the time required to receive treatment, impacts patients [7]. This study evaluates patients' perspectives of the time‐related burden of PACI treatments for PNH.

## 2 Methods

This mixed methods, cross‐sectional, observational study recruited US‐based adults with PNH who had been on PACI treatment for at least 6 months. Participants were recruited via existing databases of potential study participants and healthcare providers willing to assist with study recruitment, social media, and the Aplastic Anemia and Myelodysplastic Disease Syndromes International Foundation. Individuals were excluded if they were currently enrolled in a clinical trial, taking an investigational medicine for PNH, or had done so within the past 6 months.

Participants completed an online survey about time spent on treatment‐related activities (hours:minutes). Survey data were analyzed descriptively using SAS software (v9.4). A subset of participants then engaged in a one‐on‐one qualitative interview to discuss their experiences with PNH and treatment. Verbatim interview transcripts were coded and analyzed in NVivo (v14.0) using thematic analysis to identify and describe key elements of patients' experiences.

The study was approved by WCG IRB (#20230839). All participants provided informed consent and received a monetary honorarium for taking part in the study. Data were collected from March to September 2023, when eculizumab, ravulizumab, and pegcetacoplan were the only approved PACIs in the US.

## 3 Results

Participant demographic and clinical characteristics (survey: *N* = 61; interview: *N* = 25) are provided in Table 1.

**Table 1:** Participant demographic and clinical characteristics.

	Patients with PNH Who completed the survey (*N* = 61)		Patients with PNH Who also completed an interview (*N* = 25)	
Continuous variables (years)	Mean (SD)		Mean (SD)	
Age	46.9 (14.3)		46.0 (14.0)	
Time since PNH diagnosis	11.0 (9.3)		10.6 (8.3)	
Time since start of treatment with current complement inhibitor^a^	3.2 (2.9)		3.7 (3.7)	

**Categorical variables**	***n***	**%**	***n***	**%**
**Gender identity**				
Female	43	70.5	17	68.0
Male	17	27.9	7	28.0
Nonbinary or Gender non‐conforming	1	1.6	1	4.0
I do not wish to answer	0	0.0	0	0.0
**Race and/or ethnicity** b ^,^ c				
Caucasian or White	47	77.0	21	84.0
Black	7	11.5	3	12.0
Hispanic or Latino	6	9.8	2	8.0
Asian	3	4.9	1	4.0
American Indian or Alaska Native	1	1.6	1	4.0
Native Hawaiian or Other Pacific Islander	1	1.6	1	4.0
Other	2	3.3	0	0.0
I do not wish to answer	3	4.9	0	0.0
**Employment status** b				
Employed full‐time	20	32.8	13	52.0
Employed part‐time	13	21.3	5	20.0
Retired	13	21.3	4	16.0
Self‐employed	6	9.8	1	4.0
Homemaker	5	8.2	2	8.0
Disabled/unable to work	5	8.2	2	8.0
Unemployed and not looking for work	5	8.2	1	4.0
**Current treatment**				
Ravulizumab (Ultomiris)	37	60.7	13	52.0
Pegcetacoplan (Empaveli)	13	21.3	8	32.0
Eculizumab (Soliris)	11	18.0	4	16.0

*Note:* Responses are ordered according to frequency of the PNH survey sample except for responses of “I do not wish to answer,” which are presented last.

Abbreviations: IQR, interquartile range; PNH, paroxysmal nocturnal hemoglobinuria; SD, standard deviation.

^a^ Twenty nine survey participants and 14 interview participants reported having also received treatment with a different complement inhibitor prior to beginning their current treatment.

^b^ More than one response allowed per participant; percentage may sum to > 100%.

^c^ Race is a self‑identified social construct, not a proxy for genetic ancestry, and reflects social and structural determinants rather than biological differences.

Participants across all PACI types spent a median of 3 h and 15 min on treatment‐related activities each time they received treatment (Table 2). Participants receiving ravulizumab spent the most time on treatment‐related activities per treatment administration (3:45), while those on pegcetacoplan spent the least amount of time per treatment administration (1:15). Statistical significance was not assessed between groups. Forty percent of interviewees reported that the time associated with treatment was burdensome.

**Table 2:** Time spent on PNH treatment each time treatment is received and example quotes describing interview participants' PACI treatment‐related burdens.

	All survey participants		Patients treated with Ravulizumab		Patients treated with Eculizumab		Patients treated with Pegcetacoplan	
			Approved dosing schedule: 1 infusion every 8 weeks		Approved dosing schedule: 1 infusion every 2 weeks		Approved dosing schedule: 2 infusions every 1 week	
	*n*	Median (IQR)	*n*	Median (IQR)	*n*	Median (IQR)	*n*	Median (IQR)
**Total time spent each time treatment is received**								
Total hours spent	61	3:15 (2:15–4:55)	37	3:45 (2:55–5:30)	11	3:00 (2:35–4:30)	13	1:15 (0:45–2:25)
“I don't love the infusion day, in terms of how long it takes…it takes like, 5, 6 h by the time I get there, get the blood work done…then I have to wait an hour for the order to come…[after the infusion] they have an observation hour” (*57 years, female, ravulizumab*) “You have to do it twice a week… you have to at least allow an hour…to… warm up your medication…you have to get your supplies ready to do it and that takes another half an hour” (*62 years, female, pegcetacoplan*)								
**Time spent on specific activities**								
Preparing for treatment (e.g., scheduling, planning, coordinating help)	61	0:25 (0:10–0:30)	37	0:30 (0:10–1:00)	11	0:20 (0:05–0:30)	13	0:25 (0:10–0:30)
Traveling to and from the facility where your treatment is administered^a^	42	0:35 (0:25–1:00)	32	0:35 (0:27–1:00)	10	0:32 (0:20–0:45)	0	‐‐
Waiting or completing paperwork in the facility where treatment is administered^a^	42	0:20 (0:10–0:45)	32	0:20 (0:12–0:45)	10	0:17 (0:05–0:45)	0	‐‐
Receiving treatment (having it administered; being monitored after treatment)	61	1:30 (1:00–2:00)	37	1:30 (1:00–2:00)	11	1:15 (1:00–1:30)	13	0:45 (0:38–1:00)
Obtaining insurance authorization/approval, coverage, reimbursement, or any other activities related to insurance or payment for treatment	61	0:10 (0:00–0:30)	37	0:15 (0:00–0:30)	11	0:20 (0:00–1:00)	13	0:00 (0:00–0:10)
Other activities not described above	61	0:00 (0:00–0:30)	37	0:00 (0:00–0:30)	11	0:00 (0:00–0:30)	13	0:00 (0:00–0:00)
“Even my—my treatment schedule, having time off just to go to my treatments, I don't wanna use all my time off for health stuff. I'd like to also go on vacations, but then I feel like I'm being greedy by asking for more time off when I already have had much time off, you know” (*28 years, female, eculizumab*)“[I]t'd be nice to not have to go to the infusion center every 8 weeks…You know, it's—it interrupts my day. It interrupts my plans…I have to plan trips around that, um—it'd be nice to not have to…” (*56 years, female, ravulizumab*)“[I]f we go away for the weekend, we make sure that I'm back in time to do the infusion …[if we will not be home] they give you a cooler to take your medication in…you get worried because you have to keep it cold…[I]f we go to a hotel, we have to make sure we have a place to store the medication…plus you've got the needles, so it's not like you can travel with needles, especially on an airplane” (*62 years, female, pegcetacoplan*)								
**Time taken off work**								
Time taken off from work for each treatment among all employed patients	35	3:00 (0:00–4:30)	22	3:30 (0:00–5:00)	6	2:30 (0:00–3:00)	7	0:00 (0:00–2:00)
Time taken off from work for each treatment among employed patients who took > 0 h off	21	4:00 (3:00–5:00)	15	4:30 (3:00–6:00)	4	3:00 (2:30–4:00)	2	3:10 (2:00–4:20)
“[T]hat's one of the bigger inconveniences for me, having to actually switch my schedule around for work, I have to [drive to and from the infusion center], get the infusion done…and then I have to start work. So, it becomes a very long day very quickly” (*29 years, female, ravulizumab*)								
**Time spent by helpers**								
Time spent by a helper providing assistance with treatment‐related activities among all helpers^b^	26	2:00 (0:45–4:00)	17	2:00 (0:30–4:00)	6	2:00 (1:00–2:30)	3	2:00 (1:30–24:00)
“[R]ight after my infusion, I'm very tired, very, very tired and if I drive back, an hour and a half—and this is with really bad traffic, I'm gonna end up falling asleep…[it is difficult because] all of my kids work, my husband works…they have to decide who is able to take the day off to take me, 'cause this is an all‐day process” (*46 years, female, ravulizumab*)								

*Note:* Time is reported in Hours:Minutes, and reflects the amount of time spent each time treatment is received.

Abbreviations: IQR, interquartile range; PACI, parenterally administered complement inhibitor; PNH, paroxysmal nocturnal hemoglobinuria.

^a^ Item administered to 42 patients who received treatment at a medical facility.

^b^ Item administered to 26 patients who received help from another person.

Across the full survey sample and when stratified by treatment, receiving treatment was the most time‐consuming activity, followed by travel to/from the treatment facility, and preparation for treatment. Consistent with the survey results, interviewees treated with eculizumab and ravulizumab noted that in addition to the time associated with travel and receiving the infusion, they also spent time waiting for appointments and for infusion preparation. Two of the 8 interviewees treated with pegcetacoplan, which is self‐administered at home, reported that the time associated with the infusions was more than they would like to spend. Table 2 provides example quotes from interviews.

Thirteen interviewees (7 treated with ravulizumab, 3 with eculizumab, 3 with pegcetacoplan) noted that their ability to plan aspects of their lives (e.g., family needs, recreational activities, vacations and travel) was limited due to time associated with treatment. Participants taking pegcetacoplan found it easier to travel since they could bring their treatment, though administering it while away from home still posed challenges.

Employed survey participants (*n* = 35) reported missing a median of 3 h of work per treatment administration, especially for those on ravulizumab (3:30) or eculizumab (2:30). Interviews revealed that treatment often disrupted work schedules, requiring participants to adjust their work hours. Nearly half of survey participants reported needing help from a spouse, partner, or other family member to manage aspects of their treatment (*n* = 26, 42.6%). Helpers spent a median of 2 h assisting with treatment‐related activities per treatment administration. Two interviewees found needing help was a difficult component of treatment, and worried about burdening others.

## 4 Discussion

The findings of this study suggest the time associated with PACI treatment for PNH can be burdensome for patients. Survey results showed that participants spent over 3 h engaging in treatment‐related activities every time they received treatment. Although time associated with treatment varied by treatment type, the interference caused by treatment did not.

During interviews, participants discussed the need to plan for and around their eculizumab and ravulizumab treatment days, including not over‐scheduling in the days leading up to treatment, arranging for assistance and time off work if necessary, and planning for travel to and time at the infusion center. Between treatments, participants attempted to schedule activities like travel and vacation. Participants on pegcetacoplan reported that while it is a more convenient treatment option, there are still logistical considerations, including proper medication storage. Notably, when considering the total amount of time spent on treatment‐related activities and adjusting for treatment frequency, pegcetacoplan is the most time intensive treatment, requiring approximately 10 h over 4 weeks (assuming 2 infusions per week), compared to eculizumab at 6 h and ravulizumab at just under 2 h. While these estimations do not account for factors that may make the time spent on treatment‐related activities more tolerable, they provide useful insight into the total time‐related demands of PACI treatment.

The study results are consistent with prior research on the ways PACI treatment impacts the lives of patients with PNH. Dingli et al. found that the PNH treatment options available at the time of their study did not mitigate the impacts on patients' quality of life, leading to a call for improved treatments [8]. As seen in our study, prior research indicates that PACI treatment has a substantial impact on patients' abilities to plan their lives, as they are constantly planning around treatment [9, 10]. In their treatment preference study, Peipert et al. found that frequency of infusion was the most important factor in determining patient preference, followed by overall quality of life and patients' ability to plan activities—these factors were more important to patients than treatment efficacy [9].

This study may be limited by selection bias and a lack of demographic diversity. Those without access to social media or involvement with patient advocacy groups may not have seen the study advertisement and patients with severe symptoms may not have been able to participate. Reliance on retrospective data collection can introduce recall bias. All analyses were descriptive, and the study was not designed to test differences between participants stratified by PACI treatment.

The findings from the current study add to the body of evidence indicating that the time associated with PACI treatment is burdensome for patients with PNH. The ability to integrate qualitative data with the quantification of time associated with treatment offers a more detailed and comprehensive understanding of one critical element of living with PNH. As new treatments are developed, the findings from this study serve to support investigations into more patient‐friendly treatment administration options, potentially easing the treatment burden of patients with PNH.

## Author Contributions

**David Dingli:** conceptualization, visualization, writing – review and editing. **Lynne Broderick:** conceptualization, investigation, writing – original draft, methodology, writing – review and editing, formal analysis, project administration. **Avery A. Rizio:** conceptualization, investigation, writing – original draft, methodology, visualization, writing – review and editing, formal analysis. **Sloan Rucker:** investigation, writing – original draft, formal analysis, writing – review and editing. **Kaitlin LaGasse:** investigation, writing – review and editing, writing – original draft. **Michelle K. Carty:** conceptualization, visualization, writing – review and editing, supervision. **Elise Burton:** conceptualization, writing – review and editing. **Shaquilla Gordon:** conceptualization, writing – review and editing. **Glorian P. Yen:** conceptualization, methodology, writing – review and editing. **Jincy Paulose:** conceptualization, methodology, writing – review and editing. **Anumaxine Geevarghese:** conceptualization, methodology, writing – review and editing. **Soyon Lee:** conceptualization, methodology, writing – review and editing, supervision.

## Ethics Statement

This study was performed in line with the principles of the Declaration of Helsinki. Approval was granted by an independent Institutional Review Board (Western Institutional Review Board‐Copernicus Group [WCG], #20230839).

## Conflicts of Interest

A.G., G.P.Y., J.P., A.G., and S.L. are employees of Novartis Pharmaceuticals Corporation; G.P.Y. and S.L. also own stock in the company. S.G. and E.B. have received consultancy funding and honoraria from Novartis. D.D. has received consultancy and research funding from Novartis. L.B., A.A.R., K.L., S.R., and M.K.C. are employees of IQVIA, which received funding from Novartis to conduct this research. Several authors are employed by Novartis, the sponsor of this study. However, their employment did not influence the study's design, data collection, analysis, interpretation, the writing of the report, or the decision to submit the report for publication.

## Transparency Statement

The lead author Lynne Broderick affirms that this manuscript is an honest, accurate, and transparent account of the study being reported; that no important aspects of the study have been omitted; and that any discrepancies from the study as planned (and, if relevant, registered) have been explained.

## Data Availability

The data that support the findings of this study are available upon reasonable request to the corresponding author. The data are not publicly available due to privacy restrictions.
